# A comprehensive analysis of intraoperative factors associated with acute-on-chronic kidney injury in elderly trauma patients: blood loss as a key predictor

**DOI:** 10.1007/s40520-023-02540-6

**Published:** 2023-08-30

**Authors:** Leonard Lisitano, Timon Röttinger, Tyler Thorne, Stefan Förch, Jairo Cifuentes, Kim Rau, Panagiotis Daniel Vounatsos, Edgar Mayr

**Affiliations:** 1 Department for Trauma, Orthopedics, Hand and Plastic Surgery, University Hospital Augsburg, Stenglinstr. 2, 86156 Augsburg, Germany; 2 Department of Orthopaedic Surgery, University of Utah, Salt Lake City, UT USA

**Keywords:** Acute Kidney Injury (AKI), Chronic kidney disease (CKD), Orthopedic trauma surgery, Geriatric patients, Postoperative complications, Ortho-geriatric co-management

## Abstract

**Background:**

Postoperative acute kidney injury (AKI) is a critical issue in geriatric patients with pre-existing chronic kidney disease (CKD) undergoing orthopedic trauma surgery. The goal of this study was to investigate modifiable intraoperative risk factors for AKI.

**Methods:**

A retrospective study was conducted on 206 geriatric patients with CKD, who underwent orthopedic trauma surgery. Several variables, including intraoperative blood loss, postoperative hypoalbuminemia, intraoperative blood pressure and long-term use of potentially nephrotoxic drugs, were analyzed.

**Results:**

Postoperative AKI (KIDGO) was observed in 25.2% of the patients. The 1-year mortality rate increased significantly from 26.7% to 30.8% in patients who developed AKI. Primary risk factors for AKI were blood loss (*p* < 0.001), postoperative hypoalbuminemia (*p* = 0.050), and potentially nephrotoxic drugs prior to admission (angiotensin-converting enzyme inhibitors, angiotensin-II receptor antagonists, diuretics, antibiotics, NSAIDs) (*p* = 0.003). Furthermore, the AKI stage negatively correlated with propofol dose per body weight (*p* = 0.001) and there was a significant association between AKI and the use of cement (*p* = 0.027). No significant association between intraoperative hypotension and AKI was observed in any statistical test. Femur fracture surgeries showed the greatest blood loss (524mL ± 357mL, *p* = 0.005), particularly intramedullary nailing at the proximal femur (598mL ± 395mL) and revision surgery (769mL ± 436mL).

**Conclusion:**

In geriatric trauma patients with pre-existing CKD, intraoperative blood loss, postoperative hypoalbuminemia, and pre-admission use of potentially nephrotoxic drugs are associated with postoperative AKI. The findings highlight the necessity to mitigate intraoperative blood loss and promote ortho-geriatric co-management to reduce the incidence and subsequent mortality in this high-risk population.

## Introduction

Acute kidney injury (AKI) is a common and serious complication following orthopedic and trauma surgeries, particularly in elderly patients with pre-existing chronic kidney disease (CKD). The incidence of AKI is high in major orthopedic surgeries (5—60%), and it has a negative impact on overall outcomes, including an increased 1-year mortality [1, 2].

Comorbidities such as CKD, diabetes mellitus, and cardiac insufficiency are well-known risk factors for the development of perioperative AKI in surgical patients [3]. These factors define a vulnerable group of patients who are more likely to experience AKI following surgery. Despite the implementation of ortho-geriatric co-management, which improved the treatment of pre-existing internal diseases, perioperative AKI continues to occur—especially in this population [4, 5].

In addition, trauma surgery presents a unique challenge as surgery is often urgently required regardless of the patient's previous illnesses. Therefore, it is crucial to identify intraoperative risk factors for the development of AKI that can be actively improved during surgery.

Although some studies have examined AKI after orthopedic surgery, there is a notable gap in research specifically investigating the risk factors for acute-on-chronic kidney injury following trauma surgery in elderly patients [2, 3, 6]. Moreover, the focus on modifiable factors such as intraoperative hypotension, surgical procedures, duration of the operation, and blood loss has been largely absent. Given the limitations of the existing literature, the present study is directed towards exploring these modifiable risk factors and understanding their impact on acute-on-chronic kidney injury in elderly trauma patients.

## Methods

### Study design and participants

All patients undergoing surgery at a Level I Trauma Center between January 2021 and December 2021 were screened for inclusion in this study. Inclusion criteria were 65 years of age or older, surgery during the study period, and pre-existing CKD. Exclusion criteria were pre-existing dialysis requirement (at the time of admission), preoperative intensive care, ongoing or recently completed cytostatic therapy and pre-existing organ transplantation. For each patient, only the first surgery performed during the study period was considered. ('Revision' in the context of this study refers to surgeries performed on injuries with a preexisting post-surgery condition, such as peri-prosthetic or peri-osteosynthetic fractures.)

The primary outcome was acute kidney injury (AKI) as defined by the Kidney Disease: Improving Global Outcomes (KDIGO) criteria. [7] The secondary outcome was 1-year survival.

### Data collection

To investigate potential risk factors for AKI, data was collected on a range of variables including patient diagnoses, blood pressure during surgery (including maximum and minimum values for systolic, diastolic, and mean arterial pressure), drop in blood pressure during anesthesia, range of blood pressure, drugs used for anesthesia, dose of Propofol, duration/type of surgery, use of cement (without antibiotic infusion) and postoperative Albumin levels. Drop in blood pressure was defined as the difference between pre-anesthesia blood pressure and the lowest blood pressure recorded during anesthesia.

Medication at admission, during hospitalization and at discharge were screened for potentially harmful drugs to the kidneys such as contrast mediums, angiotensin-converting enzyme inhibitors (ACE-I), angiotensin-II receptor antagonists (AT-II), diuretics, antibiotics (aminoglycosides), non-steroidal anti-inflammatory drugs (NSAID), and opiates.

### Blood loss calculation

Additionally, blood loss during surgery was calculated using the Mercuriali formula [8]. This formula takes into account pre- and postoperative hematocrit and the number of transfused red blood cells (RBCs) as well as the patient's blood volume (BV), which is calculated by the Nadler formula [9].

For women, the formula is

$$BV (l) = height {(m)}^{3} x \,0.3561 + weight (kg)\, x\, 0.03308 + 0.1833$$

For men, the formula is

$$BV(l) = height {(m)}^{3} x \,03669 + weight (kg)\, x\, 0.03219 + 0.6041.$$

The estimated blood loss is calculated as

$${\text{BV }} \times \, \left( {{\text{Hct preop }} - {\text{ Hct day 5 postoperative}}} \right) \, + {\text{ ml of transfused RBC}}.$$

Postoperative Hct (hematocrit) values were accepted from days 4 to 6 after surgery. If necessary, (RBC) transfusions were administered based on the internal transfusion protocol, which mandated transfusions for patients with hemoglobin levels under 7 g/dl (provided the patient gave consent). Transfusions were also performed for patients with hemoglobin levels between 7 and 8 g/dl, depending on symptoms and cardiovascular risk factors.

### Data analysis

Data were collected from medical records and follow-up phone calls one-year post-surgery. These data were subsequently entered into a database for analysis using SPSS 28 (IBM SPSS Statistics für Windows. Version 28.0, IBM Germany). Information is represented as the mean ± standard deviation. The significance level was set to α ≤ 0.05, *p*-values ≤ 0.01 were considered highly significant. Exploratory data analyses were implemented to identify potential relationships between the risk factors and acute kidney failure. Differences were assessed for significance using ANOVA, Chi-Square or Fisher’s exact test, and correlations were examined using multiple linear regression and Spearman’s correlation.

### Power analysis

Prior to the data collection, a power analysis was conducted using G*Power 3.1.9.7 to determine the appropriate sample size. This was based on an estimated correlation of 0.2 between blood loss and AKI, as suggested by older data from a preliminary (in-house) study. The aim was to achieve a power greater than 80% and to set the level of significance (alpha) at ≤ 0.05. The power analysis recommended a sample size of 154. Considering the estimated annual patient eligibility of 200–300, the duration for sample collection was set at one year. The year 2021 was chosen as it was the most recent year for which complete 1-year survival data were available.

### Ethical considerations

This study complies with the ethical standards set forth in the Declaration of Helsinki and was approved by the Ethics Committee of the Bavarian State Medical Association (Bayerische Landesärztekammer) (date of approval 28.02.2023).

## Results

A total of 206 patients were included (96 males and 110 females). The average age of the participants was 82.77 ± 7.73 years, with a median age of 84 years, and an age range between 66 and 99 years. 52 of these patients (25.2%) developed an acute on chronic kidney injury within 7 days after surgery. The injuries sustained by the patients were diverse, with 40 fractures of the upper extremity, 2 pelvic fractures, 125 fractures of the lower extremity, 16 spinal fractures, and 23 other types of injuries. Overall, the 1-year mortality rate was 26.7% (55/206). For patients who developed postoperative AKI, the 1-year mortality rate was even higher, at 30.8% (16 of 52).

### Acute on chronic kidney injury

A Spearman correlation test showed a significant positive correlation between blood loss (in mL) (*p* < 0.001) and transfusion volume (in mL) (*p* < 0.001) with the AKI stage according to the KDIGO criteria. A significant negative correlation was observed between postoperative albumin levels and AKI (*p* = 0.050), indicating that lower albumin levels were associated with a higher incidence of AKI. Furthermore, a significant negative correlation was found between the induction dose of propofol (in mg) and AKI stage (*p* = 0.007), as well as between the induction dose of propofol per bodyweight (in mg/kg) and AKI stage (*p *= 0.001). There was a significant positive relationship between the induction dose of propofol per body weight and the minimum mean arterial pressure (MAP) during surgery (*p* = 0.033). No significant association between intraoperative hypotension and AKI was observed in any statistical test. Different risk factors for the development of AKI are presented in Table 1.

**Table 1:** Presents the correlation of various risk factors with the AKI stage (KIDGO), using Spearman’s Rho for the analysis. It includes factors like “minimal MAP”, which represents the minimum MAP that persisted for at least 5 min, and “MAP decrease”, which indicates the drop in MAP during the induction, “Min Systolic”, which presents the minimum systolic blood pressure that was documented for at least 5 min, and “Time Systolic”, which presents the time in minutes with a systolic pressure under the stated value, “Albumin level” represents the post-surgery serum albumin. Significant values are marked with an *, while highly significant values are marked with **

	Correlation Spearman	Significance
Blood loss	0.249**	*p* < 0.001
Transfusion volume	0.310**	*p* < 0.001
Propofol/bodyweight	− 0.239**	*p* = 0.001
1 year mortality	− 0.042	*p* = 0.549
Min MAP	− 0,034	*p* = 0.624
MAP < 60 mmHg	0.001	*p* = 0.986
MAP decrease	− 0.074	*p* = 0.305
Min systolic	0.015	*p* = 0.832
Time systolic < 80 mmHg	0.020	*p* = 0.773
Time systolic < 70 mmHg	− 0.033	*p* = 0.643
Aortic valve stenosis	0.023	*p* = 0.741
Albumin level	− 0.140*	*P* = 0.050
Risk score ≥ 3	0.290**	*p* < 0.001

Additionally, a multiple linear regression analysis was conducted, incorporating blood loss, Propofol/bodyweight, min MAP, time systolic < 80 mmHg, time systolic < 70 mmHg, and min systolic pressure. The results of this analysis yielded significant differences (*p* = 0.02), with blood loss standing out as a paramount risk factor, as indicated by a highly significant (*p* = 0.001) and an R-value of 0.309. Given that the risk score includes blood loss, albumin, and nephrotoxic medications, it is not independent of these factors. Hence, an additional multiple linear regression analysis was performed without these variables, revealing also the risk score as highly significant (*p* < 0.001, *R* = 0.338).

Chi-square test and Fisher's exact test showed a significant association between the AKI stage (KIDGO) and the use of cement (*p* = 0.027), as well as between AKI stage (KIDGO) and postoperative hypoalbuminemia (defined as albumin levels lower than 25 g/L) (*p* = 0.031). A significant association was found between the preoperative use of potentially nephrotoxic medications as part of the patients’ long-term medication regimen and the development of AKI (*p* = 0.028). Potentially nephrotoxic medications included diuretics (except Torasemide ≤ 10 mg/day), angiotensin II receptor blockers (ARBs), angiotensin-converting enzyme (ACE) inhibitors, non-steroidal anti-inflammatory drugs (NSAIDs), and nephrotoxic antibiotics.

To calculate Odds Ratios, the AKI stage was converted into the presence or absence of AKI according to KDIGO criteria. Significant associations were found for the use of cement, preoperative potentially nephrotoxic medications, high-risk patients (risk score ≥ 3), blood loss greater than 500 mL, and blood loss greater than 1000 mL (Table 2).

**Table 2:** Displays statistically significant risk factors for the development of acute-on-chronic kidney injury and the corresponding Odds Ratios. For this purpose, the data were summarized as AKI yes or no (AKI yes representing all stages of AKI according to KDIGO criteria)

	Chi-Square	Fisher´s exact	Odds ratio
Blood loss ≥ 500 mL	*p* = 0.005	*p* = 0.005	2.524
Blood loss ≥ 1000 mL	*p* < 0.001	*p* = 0.002	6.278
Medication	*p* = 0.003	*p* = 0.002	3.323
Cement	*p* = 0.013	*p* = 0.011	2.267
Risk Score ≥ 3	*p* < 0.001	*p* < 0.001	9.000

The incidence of AKI varied across different injury types, being highest for femur fractures (34.7%, 41/118) and absent for lower leg/foot fractures (0/7). An ANOVA test revealed these differences in AKI incidence were not statistically significant (*p* = 0.662).

### Blood loss

**Fig. 1:**
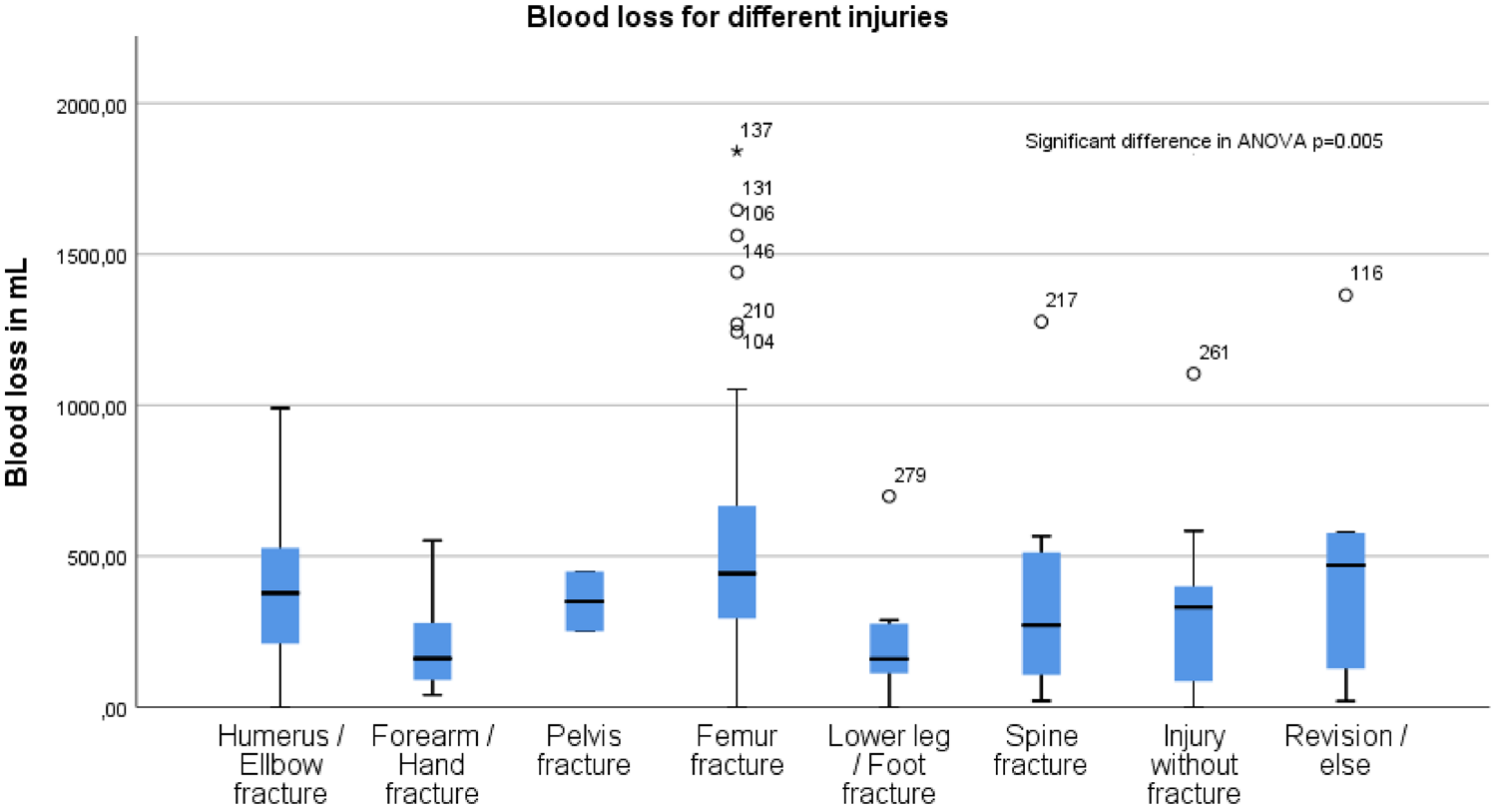
presents boxplots depicting blood loss in milliliters (mL) for various types of injuries

Blood loss varied among the different types of injuries, with the highest values observed for femur fractures (average 524 mL ± 357 mL) and revision surgeries (average 505 mL ± 477 mL), and the lowest for forearm (average 201 mL ± 150 mL) and lower leg fractures (average 233 mL ± 227 mL). ANOVA identified a highly significant difference between the groups (*p* = 0.005). Post hoc multicomparison analysis than confirmed a significant difference between forearm/hand fractures and Femur fractures (*p* = 0.044, Tukey-HSD) (Fig. 1).

**Fig. 2:**
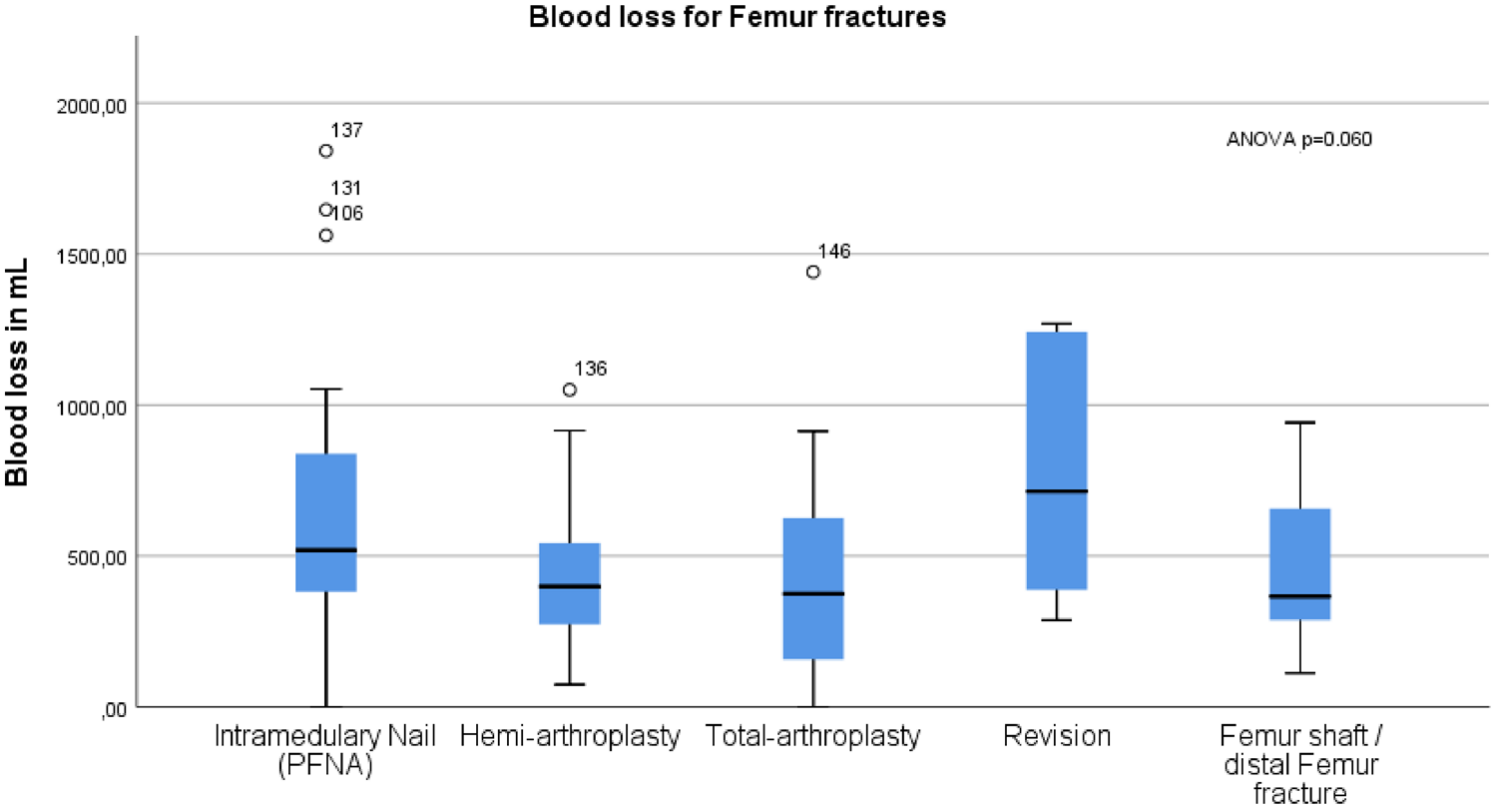
presents boxplots depicting blood loss in milliliters (mL) for femur fractures

Within the femur fractures, differences were observed, with the highest blood loss reported for revisions (average 769 mL ± 436 mL), followed by intramedullary nailing (average 598 mL ± 395 mL), hemi-arthroplasty (average 430 mL ± 243 mL) and total-arthroplasty (average 421 mL ± 337 mL)(ANOVA *p* = 0.060) (Fig. 2).

### Albumin

A significant negative correlation was observed between postoperative albumin levels and the development of AKI (Spearman's correlation coefficient—0.140; *p* = 0.050), implying that lower albumin levels were associated with an increased likelihood of AKI. Albumin levels also significantly negatively correlated with the 1-year mortality rate (Spearman's correlation coefficient—0.187, *p* = 0.011). A highly significant correlation was detected between postoperative albumin levels and the induction dose of propofol (correlation coefficient 0.230, p = 0.003). Moreover, a significant negative correlation was found between the duration of the surgery and postoperative albumin levels (correlation coefficient—0.370, *p* < 0.001), suggesting that longer surgeries were associated with lower postoperative albumin levels.

Further analysis utilizing a multiple linear regression model displayed a highly significant overall effect (*p* < 0.001, *R* = 0.445) of these factors. Within this model, 1-year-mortality (*p* = 0.009) and duration of surgery (*p* < 0.001) demonstrated highly significant associations with postoperative serum albumin levels. However, the links with acute kidney injury (AKI) (*p* = 0.110) and Propofol induction dose (*p* = 0.250) did not reach statistical significance.

### Risk score

To summarize the identified risk factors, points were assigned for blood loss, postoperative hypoalbuminemia, and the presence of potentially nephrotoxic medication at admission, and these were evaluated as risk score.

**Table:** 

Blood loss	0–500 mL	0 points
	500–1000 mL	1 point
	> 1000 mL	2 points
Hypalbuminemia	< 25 g/L	1 point
Nephrotoxic medication	yes	1 point

Patients with a score of 3 or higher were classified as high-risk patients. The score showed a highly significant association with the occurrence of AKI (*p* < 0.001) in the Chi-square and exact Fisher's test, as well as in the multiple linear regression (*p* < 0.001, *R* = 0.338). With an odds ratio of 9, these high-risk patients have a highly increased risk of developing AKI in the immediate postoperative period compared to patients with lower scores. The high-risk score demonstrated a positive predictive value of 71.4% and a negative predictive value of 78.3%, indicating its effectiveness in identifying patients at risk of developing AKI.

### Other findings

The analysis revealed a significant positive correlation between blood loss and transfusion volume (Spearman's correlation coefficient 0.222; *p* < 0.001). Lastly, a significant positive correlation was found between the duration of the surgery and the number of days by which the surgery was postponed (Spearman's correlation coefficient 0.534; *p* < 0.001), indicating that a longer delay in surgery was associated with a longer operative time.

## Discussion

In this study, the incidence and risk factors of postoperative AKI in geriatric patients with pre-existing CKD undergoing orthopedic trauma surgery were examined. A high incidence of postoperative AKI was observed at 25.2%. It was noted that the incidence of AKI significantly increased the 1-year mortality rate from 26.7 to 30.8%, a finding that aligns with the existing literature which points to a higher mortality associated with AKI [10].

The primary risk factors for AKI development in this study's cohort were found to be blood loss, postoperative hypoalbuminemia, and the long-term administration of potentially nephrotoxic drugs prior to admission. Contrary to some literature, intraoperative hypotension did not emerge as a significant risk factor for AKI in this study [11, 12].

Postoperative hypoalbuminemia is a recognized risk factor for postoperative AKI, and this study also identified a significant correlation [13]. Albumin plays a crucial role in maintaining osmotic pressure, thereby influencing the distribution of fluid between tissues, the interstitium, and blood vessels [14]. This pivotal function of albumin provides a plausible explanation for its significant impact on renal function.

Hypoalbuminemia, beyond its correlation with AKI, demonstrated a statistically significant association with higher 1-year mortality. This link is likely multifactorial, reflecting not only the impact of hypoalbuminemia on renal function but also its role as an indicator of malnutrition or severe underlying diseases [13]. These conditions can further complicate postoperative recovery and overall prognosis. In addition, hypoalbuminemia was found to be associated with extended hospital stays, increased complication rates, and a heightened likelihood of discharge to a rehabilitation facility [15]. In a cohort study of geriatric patients in general surgery, hypoalbuminemia was also identified as a significant risk factor for frailty, a clinical state that predisposes to adverse health outcomes. [15] Within surgical contexts, frailty correlates with longer in-patient stays, a greater need for specialized post-discharge care, and increased mortality rates [16, 17]. Thus, albumin levels should be interpreted not just as an indicator of renal function but also as a reflection of broader health status and frailty, underlining their integral role in the comprehensive management of elderly trauma patients.

The observed significant correlation between albumin levels and the induction dose of propofol can be rationalized considering the pharmacokinetics of propofol. Propofol is highly protein-bound, with over 98% of it binding primarily to hemoglobin and albumin [18]. Consequently, only the free fraction of propofol, accounting for 1.2–1.7%, exerts the anesthetic effect. Hence, lower serum albumin levels could potentially increase the effect of propofol, thus requiring a smaller induction dose by the anesthesiologist to achieve an adequate anesthetic effect [14, 18].

Furthermore, in this study, a significant negative correlation was observed between the induction dose of propofol and the development of AKI, suggesting that propofol may confer a protective effect on renal function. Interestingly, this possible protective effect of propofol aligns with the findings of a randomized controlled trial conducted on cardiac surgery patients, which demonstrated a protective effect of propofol in comparison to sevoflurane [19].

Indeed, postoperative albumin levels provide critical insights into the probability of AKI occurrence [13]. However, in trauma surgery, the treating physician's ability to influence postoperative albumin levels is limited. Short-term interventions, particularly applicable in urgent operations, are either through shortening the surgery duration or implementing substitution strategies. Even though the duration of surgery had a significant impact on postoperative albumin levels in this study (longer surgery leading to lower postoperative serum albumin levels), it is already standard practice to keep surgery times short in geriatric patients to overall minimize complications.

Potentially nephrotoxic medications at the time of admission are also a well-known risk factor and have been identified as a significant influence in the present study [20]. However, in the case of urgent trauma surgeries, there is no opportunity for intervention regarding these medications.

Greater opportunities for intervention present themselves with blood loss. Consistent with other publications, this study identified blood loss as one of the greatest risk factors for postoperative AKI [12]. Surgeons can actively influence blood loss through various measures, such as the choice of access and osteosynthesis, the use of a cell-saver, even in procedures with little expected blood loss, or RBC transfusion. [21] Additionally, there is the possibility of using anticoagulant medication [22]. In a large study on proximal femur fractures, Fenwick et al. were able to demonstrate a significant effect in reducing blood loss through the local and systemic use of tranexamic acid [23]. These findings are particularly relevant to the present study, as femur fractures showed the highest blood losses compared to other injuries. Among femur fractures, intramedullary nailing at the proximal femur (PFNA, TFNA) and revision surgery resulted in the greatest blood losses, consistent with the findings of Fenwick et al. [23].

The risk score used in this study, which includes blood loss, medication at admission, and postoperative serum albumin, showed good predictive values for an increased risk of acute-on-chronic kidney failure in geriatric patients. Various risk scores have been suggested in the literature to evaluate perioperative AKI risk [24, 25]. However, merely identifying at-risk groups does not lead to a reduction in the incidence of perioperative AKI. In particular, geriatric patients with pre-existing CKD already have an indication for kidney-protective fluid management and increased awareness for renal and cardio-vascular complications, regardless of risk scores.

Through the now widespread implementation of ortho-geriatric co-management, optimal postoperative care and early detection of AKI should be generally ensured [4, 26, 27]. Consequently, patient care can be substantially improved through prevention or pre- and intraoperative interventions. The focus of the present study, therefore, was on perioperative factors that can be actively influenced, such as blood loss.

Despite numerous publications on perioperative AKI risk, to our knowledge, no study to date has specifically addressed the risk group of geriatric patients with pre-existing CKD in trauma surgery [2, 6, 12, 20]. This study attempts to fill that gap in the literature. However, trauma patients are at particular risk, as the urgent nature of the surgery often allows little time for preparatory measures, and falls (leading to fractures) frequently affect severely ill elderly patients. It is precisely these patients who benefit from a reduction in complications through directly implementable measures. This study has identified intraoperative blood loss as an essential risk factor in the multifactorial development of perioperative acute-on-chronic kidney injury. Importantly, this factor is directly manageable, offering a practical avenue for risk reduction [23, 28, 29].

The multifactorial nature of AKI poses an inherent challenge to this study, as it entails numerous confounding factors. Despite our best efforts to meticulously test and evaluate potential risk factors, as informed by the existing literature, a complete exclusion of further influences remains elusive in this retrospective study design. The correlations observed in this study were mathematically defined as “weak”. Nevertheless, they are of significance as they were either statistically significant or highly significant. Importantly, given the multitude of factors contributing to AKI, such “weak” correlations were to be expected. Moreover, these findings align with the existing body of knowledge and literature, reinforcing their scientific validity and relevance. Unfortunately, due to the retrospective design, it was not possible to gather adequate data on the further course of CKD for enough patients. This would be highly interesting, especially in light of the mutual influence of AKI and CKD [30]. Therefore, further prospective investigation is necessary. Additionally, it would be interesting to explore the impact of measures to reduce intraoperative blood loss on the prevalence of AKI in these patients. This also necessitates a prospective, ideally randomized, study design.

Strengths of this study include its focus on a highly relevant patient group with a high risk of complications and a high 1-year mortality rate, as well as its emphasis on modifiable parameters relevant for treatment. Despite the stringent inclusion and exclusion criteria, a sufficient case number was achieved to investigate various influencing factors. Furthermore, the study was able to identify intraoperative blood loss as a key factor for the development of perioperative AKI in elderly trauma patients with preexisting CKD.

In summary, for patients at high risk of developing perioperative AKI, we recommend choosing surgical procedures with the least anticipated blood loss and implementing measures to reduce blood loss, such as the local and/or systemic use of tranexamic acid. Furthermore, ortho-geriatric co-management is advisable to ensure adequate postoperative care—especially focusing on early diagnosis and treatment of complications like AKI.

## Conclusion

Blood loss, postoperative hypoalbuminemia, and potentially nephrotoxic medications at admission are the strongest risk factors for the development of acute-on-chronic kidney injury in geriatric trauma patients. Blood loss has proven to be a modifiable and statistically highly significant influencing factor. Therefore, we recommend—especially in this patient clientele with a high risk of developing perioperative AKI—to take all available measures to keep blood loss as low as possible.

## Data Availability

The datasets used and analysed during the current study are available from the corresponding author upon reasonable request.
